# How might the public contribute to the discussion on cattle welfare? Perspectives of veterinarians and animal scientists

**DOI:** 10.1017/awf.2023.88

**Published:** 2023-10-12

**Authors:** Beth Ventura, Daniel M Weary, Marina AG von Keyserlingk

**Affiliations:** 1 Animal Welfare Program, Faculty of Land and Food Systems, 2357 Main Mall, The University of British Columbia, Vancouver, BC, Canada, V6T 1Z4; 2 Dept of Life Sciences, University of Lincoln, Lincoln, Lincs LN6 7DL, UK

**Keywords:** animal welfare, beef cattle, dairy cattle, focus group, public communication, social sustainability

## Abstract

Veterinarians and animal scientists can provide leadership on issues relevant to farm animal welfare, but perceptions of these stakeholders regarding societal expectations for welfare are underexplored. This study involved five focus groups of veterinarians and animal scientists (n = 50 in total), recruited at a European meeting focused on cattle welfare. Participants were invited to discuss topics related to cattle welfare and were prompted with questions to elicit their perspectives of public concerns and how the participants felt public input should be included when developing solutions. Discussions were moderated by trained facilitators, audio-recorded and transcribed, and transcripts analysed using reflexive thematic analysis. Ultimately, four primary themes were developed: (1) The public as concerned; (2) The public as ignorant; (3) The public as needing education; and (4) The public as helper or hindrance. Groups identified specific farming practices viewed as concerning to the public, including lack of pasture access, behavioural restriction, and painful procedures. Discussions about these concerns and the role of the public were often framed around the assumption that the public was ignorant about farming, and that this ignorance needed to be rectified through education. Participants were generally ambivalent in their beliefs regarding public contributions to solutions for farm animal welfare but suggested that consumers should pay more for products to help shoulder any costs of welfare improvements.

## Introduction

The livestock sectors face ever-growing scrutiny regarding the care of their animals (Shields *et al.* 2017). While beef and dairy production appear to be perceived as conferring better care than other sectors (i.e. laying hens, pigs), social concern about the welfare of animals in these systems is increasingly well-documented (see Alonso *et al.* 2020). When asked what constitutes high standards of animal care, those without direct links to animal agriculture (e.g. public citizens and consumers) often highlight elements related to natural living, such as outdoor access, space, and behavioural freedom (Velde *et al.* 2002; Bock & Van Huik 2007; Vanhonacker *et al.* 2007). In contrast, those directly involved with animal agriculture (e.g. farmers, veterinarians) tend to emphasise health and production (i.e. biological functioning, see Balzani & Hanlon 2020). These differences in weighting given to the different elements of animal welfare (Fraser *et al.* 1997) contribute to a disconnect between societal values and industry practices (Alonso *et al.* 2020) and may hinder those in agriculture from taking public concerns seriously. Growing evidence suggests that people working in agriculture and the animal and veterinary sciences sometimes question the legitimacy of public concerns about farm practices, in part because they believe that the public knows little about these practices (e.g. Dutch pig farmers [Benard & de Cock Buning 2013]; Brazilian dairy farmers, veterinarians, nutritionists, and agronomists [Cardoso *et al.* 2019]; American veterinary students [Dolby & Litster 2019]; Australian sheep and cattle farmers [Buddle *et al.* 2021]; American animal science students [Ritter *et al.* 2021]). In doing so, such stakeholders may adopt a deficit model mindset with respect to public engagement (Simis *et al.* 2016); that is, they discount or dismiss public concerns about animal farming because they perceive these concerns to be rooted in ignorance.

Disagreements between the public and those who arguably have greater understanding of the animal industries can contribute to the loss of trust and may ultimately erode the very relationships necessary to resolve challenges to animal welfare. Some have described certain farm animal practices as ‘wicked problems’ Bolton & von Keyserlingk 2021) – where the issue is socially complex; where causes, nature, and solutions are uncertain, contested, and multifaceted and for which the involved actors diverge in views and values (Rittel & Webber 1974; Head 2008); and where solutions will require transformational change rather than implementing a technical solution. An important step in resolving complex challenges involving different groups is to identify areas of agreement before moving on to more contentious topics (Rutledge 2009). For example, it has been suggested that animal welfare challenges may best be approached through establishment of multi-stakeholder collaborative networks (Fernandes *et al.* 2019). Multi-stakeholder networks are an example of adaptive, participatory, and transdisciplinary approaches (Head & Xiang 2016) and are characterised by the fostering of discussion among a diverse set of stakeholders and collaborative knowledge-sharing. Critically, Fernandes *et al.* (2019) advised that it is important to incorporate voices from industry, academia, and the community (i.e., the public) from the outset, and that establishing trust amongst these groups is critical if they are to establish a shared sense of purpose. However, establishment of trust among such groups rests at least partially on all members being able to recognise the legitimacy of others at the table.

Attention to voices from the veterinary communities is of particular importance, as veterinarians are perceived as leaders in animal welfare (Dawson *et al.* 2016; Dolby & Litster 2019), play important roles in advising and supporting improvements in farm animal health and welfare (Croyle *et al.* 2019), and help shape evolving professional norms through their training of future generations (Ritter *et al.* 2021). While attempts have been made to investigate how those working within the livestock industries, including veterinarians, understand cattle welfare and their role in resolving problems (Ventura *et al.* 2016b; Wynands *et al.* 2021), less attention has been paid to their perspectives on the inclusion of public voices. Such information would yield valuable insight into how to develop effective multi-stakeholder networks. This study aimed to address this gap, using focus group discussions with cattle veterinarians and scientists to explore their perceptions of public concerns about cattle welfare and roles the public may play in improving cattle welfare.

## Materials and methods

### Ethical approval

The University of British Columbia Behavioural Research Ethics Board approved this study under protocol #H12-02429.

### Focus group approach and participants

A full description of the focus groups, participants and their perspectives on cattle welfare challenges and solutions can be found in Ventura *et al.* (2016b). In brief, attendees to the 7th Boehringer Ingelheim Expert Forum on Farm Animal Well-Being in Madrid, Spain (summer 2014) were invited by conference organisers to participate in focus groups about stakeholder roles in addressing challenges to cattle welfare. Focus groups were selected to allow inter-stakeholder engagement and elicit in-depth perspectives (Albrecht *et al.* 1993; Carey & Smith 1994).

All forum participants (n = 50, predominantly European [84%] and male [80%]) provided their written consent and joined one of six groups ranging from 7–9 people each in keeping with recommendations for focus group research (Krueger & Casey 2009). Four of the groups were composed primarily of veterinarians whose practices included beef and/or dairy cattle (‘Veterinarians’); the remaining two groups were a mix of veterinarians and animal scientists: one consisted mostly of academic veterinarians (i.e. veterinarians with university appointments and involved in teaching and research; ‘Academic Veterinarians’), and one with primarily animal scientists working in academia or industry (‘Animal Scientists’) (Table 1). Discussions were led by trained moderators (all authors and two individuals facilitating the conference) and lasted approximately 75 min. Groups were audio-recorded and these recordings were transcribed *verbatim* by a professional transcription service (Duly Documented Transcription Services, CA, USA).

**Table 1.** Profession, gender, and country of residence reported by participants in each of the five focus groups

	Group A	Group B	Group C	Group D	Group E
Profession	8 veterinarians	8 veterinarians	4 academic veterinarians	1 veterinarian	7 veterinarians
			5 animal scientists	1 academic veterinarian	
				6 animal scientists	
Gender	2 women	2 women	3 women	6 women	7 men
	6 men	6 men	6 men	2 men	
Country	Belgium	Belgium	Canada	Canada	Belgium
	France	France	France	Germany	Italy
	Germany	Germany	Germany	Netherlands	Netherlands
	Netherlands	Ireland	Spain	New Zealand	Sweden
	Turkey	Italy	Sweden	Spain	Turkey
	United Kingdom	Netherlands	United States	United Kingdom	United Kingdom
		Romania			
		United States			

Questions and resulting discussion related to participants’ views regarding cattle welfare issues and their own roles in resolving these were described in earlier work (Ventura *et al.* 2016b). This paper now focuses on our secondary aim, which was to elicit our participants’ views of the public’s role in addressing welfare issues. To stimulate discussion in this area, participants were asked two primary questions during the focus groups: (1) *“Should the public have a role in helping to address cattle welfare issues? If so, how?”* and *(*2) *“What do you think are the public’s key concerns about cattle welfare, and to what extent are these concerns legitimate?”* A probing question, “*Can you comment on what you think the public knows about dairy farming, and how this relates to their concerns?”* was asked after the second question if participants had not already raised the topic of public knowledge about farming.

### Qualitative analysis

We sought to find patterns of meaning across our data that described how our participants approached the question of ‘the public’ – thus we pursued a form of reflexive thematic analysis to identify, describe and interpret meaningful patterns in the data (see Braun & Clarke 2021) as follows: BV checked transcripts for accuracy against the original audio and read and re-read the transcripts to become familiar with the data. As it became apparent that participants sometimes raised the topic of ‘the public’ (e.g. referring to consumers, or lay citizens) before the two questions were asked; the full transcripts were analysed to capture how participants constructed meaning around public concerns and roles. Our approach was deductive in that our question guide and study aim sought to elicit participant perspectives on specific issues in which we were interested, but ultimately the transcripts were coded inductively to ensure that the themes were driven by the data (Braun & Clarke 2006). Transcripts were analysed first within each focus group discussion, with BV assigning codes and sub-codes to related sections of text until a preliminary codebook (list and description of themes) was created for each group’s transcript. BV kept notes of the emerging reflections as the data were processed (Miles *et al.* 2014). Lists were then compared to generate a single codebook to encompass themes across groups; this was then reviewed and modified. A final codebook was obtained after back-checking through all transcripts and after discussion with MvK. Supporting quotes are embedded in the discussion of themes to ensure participants’ voices remain centred and to support transparency of the research process (Roller & Lavrakas 2015). Quotes are labeled by participant identification to designate role, focus group and participant number (e.g. ‘Veterinarian A1’ indicates a practicing veterinarian from Group A).

### Reflexivity statement

Researchers are individuals, with worldviews that inform how they approach their subject matter, their participants, and their pursuit of scientific research; acknowledging how our own perspectives and experiences may influence our work can help contextualise the research and ultimately benefits research transparency (Holmes 2020). BV completed her PhD in the UBC Animal Welfare Program and has held animal welfare science faculty appointments in the US and the UK. DMW and MvK are Professors in the UBC Animal Welfare Program and have collaborated extensively for over two decades, addressing questions on how to improve the lives of animals under human care using different approaches and methodologies adapted from both the natural and the social sciences. As animal welfare scientists our perspectives are informed by our training and experiences and we may have held shared experiences and beliefs with some of our participants, especially those working in positions where research is undertaken.

## Results and Discussion

**Figure 1.**
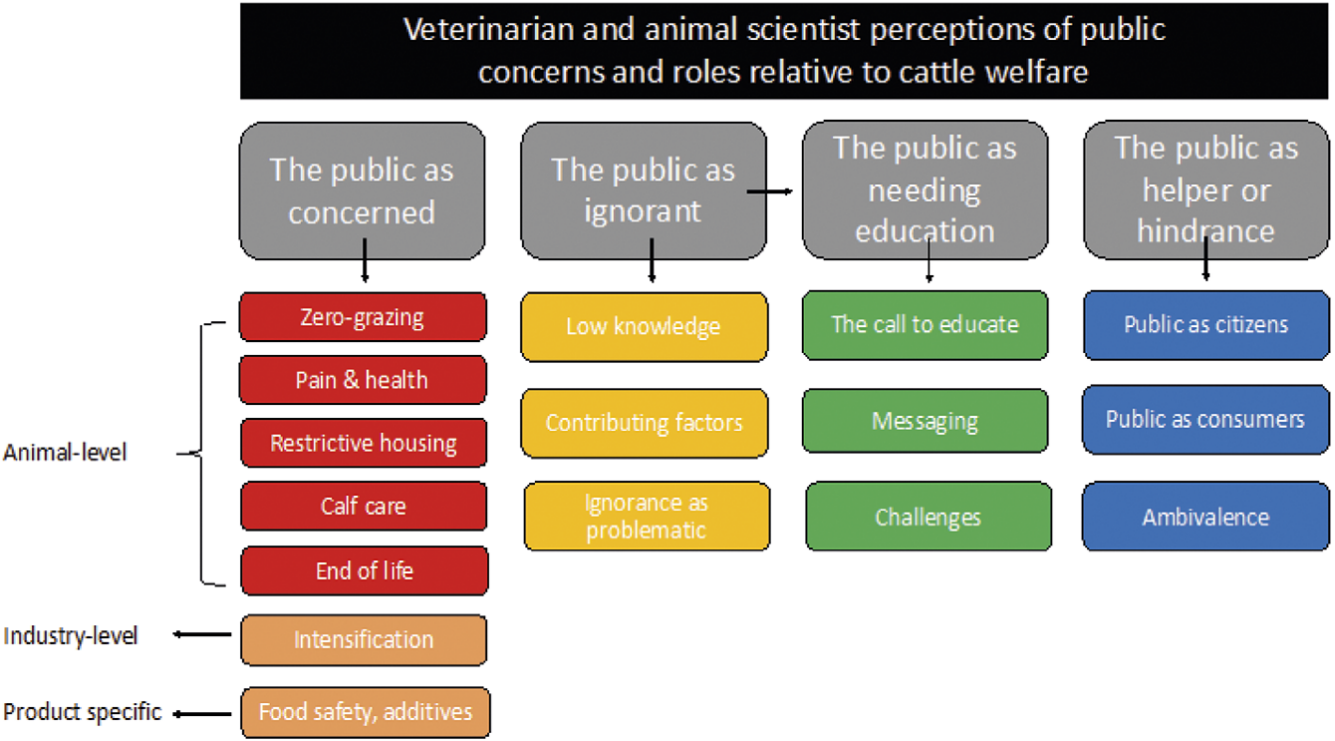
Thematic map of themes and sub-themes arising from five focus group discussions with 50 veterinarians and animal scientists, addressing the role of the public in contributing to cattle welfare discussions. The large grey boxes represent the overarching themes, and the smaller coloured boxes indicate sub-themes. Arrows are used to demonstrate linking of themes, e.g. participants’ perceptions of the public as ignorant influenced their desire for public education.

Four primary themes were developed from our analysis: (1) The public as concerned; (2) The public as ignorant; (3) The public as needing education; and (4) The public as helper or hindrance (Figure 1). All but one of the focus groups (Group E) voiced their views about the public without prompting (i.e. before being presented with the questions described above), suggesting that this topic was top-of-mind and influenced participants’ broader views regarding cattle welfare.

### Theme 1: The public as concerned

Participants were prompted to discuss the welfare concerns of the public, but groups varied in how they responded. Participants in two groups (C and D) framed their comments by acknowledging that ‘the public’ consists of many different types of people with a wide variety of views; this perspective is consistent with research showing, for example, that social concerns about animals and their welfare differ by geographical region, nationality, and socioeconomic status among other factors (Busch & Spiller 2016). All groups suggested that the public worried about animal-level issues, while some also pointed to overarching concerns affecting the livestock industries, as well as to the food products generated. These concerns are discussed in the following sections.

### Animal-level concerns

Issues highlighted were organised across five areas: pasture access; pain and health; restrictive indoor housing; calf care; and longevity and end of life.

#### Pasture access

Participants in every group identified the lack of pasture access as an area of societal concern, suggesting that the public desired to see cows living on pasture and that zero-grazing systems provoked concern. For example, Veterinarian B8 commented that, “*the consumer … will buy milk from a company that he sees the cows in the pasture, he will be so happy it’s all bio* [organic]*, it’s all natural, it’s all green.*” That participants identified the lack of access to pasture as a concern is not surprising given that research on public views of dairy farming consistently reports this result (e.g. in Canada [Schuppli *et al.* 2014]; Brazil [Hötzel *et al.* 2017]; the UK [Jackson *et al.* 2020]; and Australia [Hendricks *et al.* 2022a]). Moreover, valuing pasture access is not unique to the public, as farmers and veterinarians also express beliefs in the importance of pasture and outdoor access (Schuppli *et al.* 2014; Smid *et al.* 2022).

#### Pain and health

Four of the five focus groups (all but Group A) cited lameness and other painful conditions or procedures (e.g. routine procedures like castration and branding) as likely sources of concern, especially if public awareness of these issues was to increase. For example, in keeping with increasing consensus among stakeholders like farmers and veterinarians on the importance of lameness (Huxley 2012; Sadiq *et al.* 2019), our participants felt that lameness was one of the most important issues for the public (e.g. “*Lameness in dairy cows in the UK, the general public has zero tolerance*” [Veterinarian E3]) or something that, if known, would prompt public concern (“*If the consumer knew that in Ireland 25% of cows were lame walking to the fields, they’d say, how to stop that?*” [Veterinarian B6]). That lameness is considered a welfare problem by stakeholders both within and external to the dairy sector is not surprising. For instance, in some of our previous focus group work (Ventura *et al.* 2014) veterinarians, farmers and other dairy professionals identified lameness as a key welfare concern facing the dairy industry. However, the dairy sector continues to be challenged by the identification and treatment of lame cows in need of care (Jensen *et al.* 2022), despite increasing attention to and awareness of lameness as a production and welfare challenge by dairy stakeholders (Wynands *et al.* 2021).

#### Restrictive housing

Participants in two focus groups (B and D) discussed public concerns associated with the use of housing perceived to be intensive or restrictive of behavioural freedom (e.g. feedlots in the beef industry, tie stalls in the dairy industry). For example, Veterinarian B2 shared that the public focused on “*things that relate more to how animals are housed*: *free stall barns or feedlots*…” Similarly, Animal Scientist D1 suggested that “*from the cow’s perspective, in a tie stall, she has no freedom whatsoever … she can lay down, that’s the only thing she can do. So, I think from that point of view, the public really doesn’t like tied up cows.*” Considerable previous research has also shown that management practices that restrict movement are questioned by the public. For example, one study reported that 65% of participants from the United States were willing to vote for a ban on tie stalls (Robbins *et al.* 2019), and research of public views toward other animal industries (e.g. pigs - Vandresen & Hötzel 2021 and laying hens - Ochs *et al.* 2018) indicates that behaviourally restrictive housing of farmed animals is socially objectionable. Evidence that at least some working within the animal sectors do not support systems that restrict the movement of farm animals was provided by Yunes *et al.* (2018), who reported that 69% of 173 respondents who were affiliated with the pig industry rejected gestation stalls.

#### Calf care

Discussions also included calf care in two (Groups B and D) of the five groups (e.g. “*I think calves* [are] *the most emotive thing*”). Participants described calf transportation, high calf mortality rates, the fate of the bull calf, euthanasia of calves, and cow-calf separation as issues that are particularly challenging. For example, Animal Scientist D1 commented about cow-calf separation, “*I think the dairy industry likes to keep it under the radar because people don’t know. I think that would be an issue if it got out.”* There are now several studies indicating that issues associated with calf care, particularly in the dairy industry, prompt societal concern (Placzek *et al.* 2020). Recent work indicates that there may be growing acceptance by veterinarians working within the cattle industries that public concern in this area should not be discounted. For example, Hendricks *et al.* (2022b) interviewed Canadian veterinarians about the fate of surplus calves and reported that they felt that they had a role in educating their clients as to the changing societal norms around animal care.

The issue of dairy cow-calf separation may be especially challenging to resolve given that the practice prompts public concern (Boogaard *et al.* 2008), but many farmers perceive early separation to be beneficial and express reluctance about extended contact (Wagenaar & Langhout 2007; Ventura *et al.* 2013; Pempek *et al.* 2017; Neave *et al.* 2022; Hansen *et al.* 2023). However, there is also evidence that this reluctance is not universal, with some farmers reporting positive experiences with cow-calf contact systems (Hansen *et al.* 2023). Given the adoption of cow-calf contact systems by some farmers in some regions, research is needed to identify best practices that work for the cow, her calf and the farmer (Johnsen *et al.* 2016).

#### End of life

Two of the focus groups also discussed end-of-life issues, including transportation, cow longevity, and slaughter. For example, Animal Scientist C5 shared that, “*The consumers have a lot of problems with … the slaughterhouse process … always this issue about the killing process. And I think that’s a really important point for consumers … probably because they don’t know what happens inside.”* Academic Veterinarian C5 reflected about the relatively short lifespan of dairy cows: “*I would love to know how you could ever convince a consumer that that is a good thing … I think it’s fundamentally one of those things that most people would consider wrong at so many levels. It would be hard to construct an argument that this is a good thing.”* That our participants perceived that shortened lifespans would be of concern is unsurprising, as others have reported similar findings. For instance, Hendricks *et al.* (2022b) found that veterinarians questioned the practice of early life euthanasia of dairy calves based on the potential outcry by the public. In another study, Ritter *et al.* (2022) found that killing dairy calves was viewed as much more concerning if there was no clear purpose to their death (such as contributing to the meat supply).

### Industry level concerns

Two groups briefly described public concerns about industrial-scale farming, suggesting that the public perceives cows as living in a *“small herd”* [Animal Scientist D8]. In the words of one Academic Veterinarian (C7), “*I think a big perception for consumers, a big issue, is the factory farming issue … they perceive bigger is worse.*” Previous research from our group has shown public perspectives about the ‘ideal dairy farm’ do include a preference for smaller farms (Cardoso *et al.* 2016), but the relationship between farm size and animal welfare is complex: while larger farms may have less human-animal contact and may be less likely to provide access to pasture, they are also more likely to have improved access to technology, facilitating tracking of individual animals and early identification of animals at risk for disease (Robbins *et al.* 2016).

### Product-specific concerns

Three focus groups (B, D, and E) discussed public perceptions about the safety and quality of animal products. In the words of one veterinarian, “*… in Belgium the consumer is more interested in safe products. No drugs in it. But they pay more for it, but not for animal right[s]*” [Veterinarian E2]. Some also briefly discussed public concern over the use of hormones and antibiotics on farms. Though this commentary focused predominantly on direct (perceived) consumer safety concerns, the attention to antibiotics use is critical given the threat of antimicrobial resistance (World Health Organisation [WHO] 2021) and associated societal impetus toward responsible stewardship by the livestock industries (Clark *et al.* 2016; Busch & Fischer 2018).

### Theme 2: The public as ignorant

In each of the five groups, discussion of public concerns was framed by the belief (expressed by some members but not all) that the public was largely unaware, uneducated, and/or misinformed about animal care on farms.

#### Low knowledge

Although participants occasionally mentioned that public awareness of farm animal welfare was high (e.g. *“I think the consumers are very aware of what’s going on…”* [Veterinarian B6]), most suggested that people lacked awareness of – and specific knowledge about – farm conditions and production processes. For example, Veterinarian E3 shared, “*I think people in the UK … are very abstract about where milk comes from … they have no idea,”* a point in agreement with Animal Scientist D3: “*In the UK … actually that knowledge base is pretty low within the consumer … there’s really very little perception of what the problems are.”* Similar statements were made in other groups, e.g. *“The consumer* [doesn’t] *understand the problem of the welfare because they don’t understand the situation of the cow in a farm”* [Veterinarian B1].

Previous work has noted that people working within the livestock sectors often believe that the public is ignorant of farm procedures (e.g. UK pig producers [Hubbard *et al.* 2007]; Finnish dairy and pig producers [Kauppinen *et al.* 2010]; Canadian beef producers [Spooner *et al.* 2012]; Dutch pig producers [Benard & de Cock Buning 2013]; Australian beef and sheep producers [Buddle *et al.* 2021]; Brazilian pig producers [Albernaz-Gonçalves *et al.* 2021]; American veterinary and animal science faculty [Heleski *et al.* 2006]; American veterinary and undergraduate animal science students [Dolby & Litster 2019; Proudfoot & Ventura 2021; Ritter *et al.* 2021]). However, public understanding of animal welfare and farming practices is mediated by a range of socio-demographic factors (see Clark *et al.* 2016; Cornish *et al.* 2016; Evans & Miele 2019). When members of the public are asked to self-assess their level of knowledge, many report that they know little about production methods (Frewer *et al.* 2005; Boogaard *et al.* 2006; Ellis *et al.* 2009; Cummins *et al.* 2015; Eurobarometer 442 2016; Cardoso *et al.* 2017); to some extent this may even reflect willful ignorance (McKendree *et al.* 2014; Bell *et al.* 2017). However, when people are directly tested on their knowledge, a more complex picture often emerges; for example, in a previous study we found that public participants often already knew about lack of pasture access and other housing practices for dairy cattle (Ventura *et al.* 2016a). More empirical work is required to better document gaps in public knowledge and explore the implications of this on engagement between the cattle sectors and society.

#### Contributing factors

Like other studies of industry stakeholders’ perceptions toward the public, our participants attributed public ignorance to distance from farming, lack of education, and influence by messaging from unreliable sources (Albernaz-Gonçalves *et al.* 2021; Buddle *et al.* 2021). For example, Veterinarian E5 shared, “*No one has been to a farm, or they went to a farm when they were seven years old with school, and they can’t remember anything,”* a concern shared by Academic Veterinarian C1: “*I think for me … as a professor in a veterinary university … one of the problems* [is] *that most of my students came from the city and had absolutely no idea about how* [a farm works]*.”* Another participant [Veterinarian A8] stated: *“they don’t evaluate the behaviour of the cows because of lack of … scientific information. They* [are] *only sensitive to the marketing message.*”

#### Ignorance as problematic

The focus groups did not share a unified vision of the public. Some participants described the public as idealistic but uninformed, claiming that they wished for an unrealistic agrarian ideal that was impossible to attain given the realities of modern farming. This perspective was sometimes discussed with a sort of resigned paternalism: “*I think the general public in Sweden still lives with this romantic picture of cows at pasture, without knowing that in Sweden* [the cows] *go out for maybe four months a year, and that’s a good farm … I mean, we have the commercials on TV: Nice, sunny day, mother cow grazing, baby calf there playing. And then you get milk*” [Veterinarian E5]. Others described a public who was critical of farm practices due to specific misconceptions (e.g. “*Cows are fed antibiotics to give milk”* [Veterinarian E2]), an issue which elicited frustration amongst our participants.

Descriptions of public ignorance were sometimes used to diminish social concerns as emotional and anthropomorphic, e.g. “*The consumer’s view is very … emotional”* [Academic Veterinarian D4] and *“They often make projection[s] of human welfare to animals. That’s the big issue …”* [Veterinarian B4]. Indeed, some worried about the threats posed by public concerns: “*I think it goes back to the definition of welfare because if we allow the consumer to define that, they may well take a view that might not – that might actually create or insist that many farming systems in the world aren’t welfare-friendly and therefore shouldn’t exist”* [Veterinarian B6].

Although contributions to this discussion often framed low public knowledge as problematic (*“I think already the public is a big problem … The public* [doesn’t] *know the cow anymore …*” [Veterinarian A8]), others highlighted how the cattle industries may also benefit from low public awareness, a benefit which may wane with time. In the words of Animal Scientist C9, *“Sometimes I sort of feel a slight fear in the industry, that people will start to realise that the steak is an animal, or the milk you drink is actually representing a live animal because we as an industry tend to fear that consumers would turn into vegetarians or stop drinking milk or whatever. I don’t know, is that just me? Or is that something you experienced at all?”* [to voiced agreement]. Similarly, Animal Scientist D1 pointed out, *“It’s just that I think the dairy industry likes to keep it* [cow-calf separation] *under the radar because people don’t know. I think that it would be an issue if it got out.”* Here, rather than positioning the public in opposition to those ‘in the know’, our participants believed that views might converge if the public was privy to similar information.

### Theme 3: The public as needing education

#### The call to educate

While some groups ventured that the cattle sectors should attempt to better understand the public, a stronger consensus emerged that instead, the public should better understand farming. The need to increase or improve education efforts was largely framed as a corrective measure to rectify ignorance and to counteract exposure to messaging from other sources. For example, one participant stated, “*I think that maybe a first step* [is] *to actually teach people where food comes from*” [Animal Scientist D5] and another said “*I think the public must receive objective information*” [Veterinarian A8]. Participants were not always clear on who they felt would be best placed to lead education efforts, but some felt that they had a responsibility to assume this role. For example, Veterinarian E5 stated: “… *of course the consumers need to get information. It’s like everything in society … as vets* [who] *are working with animals … we need to put out information in a good way, at least understandable way* … [later reiterating:] … *people with information, with knowledge, we need to educate.*” The idea that public education would increase acceptability of farm practices was also voiced by sheep and cattle farmers in Australia (Buddle *et al.* 2021); however, that study’s authors also recommended that rather than relying on one-way education, the industries instead focus on building shared understandings between the public and farmers.

#### Messaging

Though unprompted, participants offered diverse suggestions to improve educational efforts, including strategies for message framing and delivery, potential education partners, and populations to target. Some participants believed that messaging should be founded in science while acknowledging the complexities of modern farming, e.g. “*I think that a very important issue also is to communicate to the consumer, to say okay, maybe it’s not perfect. But it’s not so bad* [either]*, and it’s quite good*” [Academic Veterinarian C1]. Many were emphatic about providing context for farming practices, i.e. ‘explain the why’s’, as seen in the following exchange:

*People get upset about ‘the what’ sometimes, but if they actually understand ‘the why’ … I think if you actually do have a conversation with consumers, which we often don’t do, but if you actually tell them, you know, why we do this, and this is why we do it, then it maybe helps with the discussion a little bit … they don’t get as upset because they actually understand ‘the why.’* [Animal Scientist D8].

[later in conversation]

*I think somebody was saying about ‘the why’ … people sometimes are reasonably pragmatic about that. I remember leading a group around our farm open day, and we got to the bit where we had to say, ‘and now we take the calves away’ and I thought, ‘Oh Lord, you know, I’m going to create a lot of vegans right now’* [group laughter] *… And nobody said anything. And I thought, oh, okay then. People were okay.* [Animal Scientist D1].

*I’ve had that conversation with my mother, actually* [group laughter] *… because she thought it was horrific, of course … and I kind of said to her, ‘Well, it’s actually less stressful … if you take the calf away right away than if you let them bond,’ and people have done research so then you can say it’s evidence-based which people like … And she kind of went, ‘Oh, okay.’* [Animal Scientist D8].

Participants shared stories of success in connecting with members of the public through farm visits, often with a focus on children (e.g. *“we do some local education work with schools in our area … we do farm tours,”* [Veterinarian E3]). Take, for example, the following:

*We try to do a project like this in Romania. We invite our consumers. My farm belonged to a dairy factory, and we invite kids from kindergarten till adults and all our consumers. Every week they come and they travel to the farm to see how the cow … is eating, how she is giving milk, what’s happening. And you will see that in an increase of buying our products because they understand what we are saying … we did it, because you have many kids that, when you ask them from where the milk comes or from where their mum buys the milk and she will say, the supermarket … We are living in a world now that – I had luck to see a cow, to see a chicken when I was growing up – You have kids that are growing in four walls in buildings, and they don’t see anything. And they are your future buyers.* [Veterinarian B8].

Farm visits as a form of public engagement appear to be particularly valued by those with connections to farming (Shortall & Lorenzo-Arribas 2022), perhaps because the transparency of tours is believed to improve public trust and lead to increased milk consumption, as highlighted by Academic Veterinarian C7: “*There is a dairy in northern Indiana, they milk thousands and thousands of cows. And they have what they call the glass window or glass farm, and people sit there for hours and watch cows deliver … But it’s well received, and I don’t think anyone leaves that dairy not drinking milk. In fact, they probably want to drink more of it.”*

#### Challenges

Calls to educate the public are consistent with the knowledge deficit model of public understanding (known also as the knowledge gap, informational deficit, and cognitive deficit (Wynne & Irwin 1996; Einsiedel 2000). The deficit model assumes that public concerns about science and technology are unfounded, and that experts sharing information can counteract these concerns (see Hansen *et al.* 2003). However, the deficit model is a poor foundation from which to approach public engagement about controversial topics, as numerous factors in addition to factual knowledge can influence attitudes. Given their diverse social and cultural experiences, people differ in what they value and hence what they consider acceptable in terms of animal use, and education about ‘the why’ appears to be unlikely to change people’s values. Although there is limited evidence that learning more may improve public acceptance of farming (Ferris *et al.* 2016; Smith & Ferris 2016), several studies have shown that educational efforts, including through virtual and live farm visits (Boogaard *et al.* 2011; Ventura *et al.* 2016a; Schütz *et al.* 2022), fail to meaningfully shift public acceptance of farming practices (Hötzel *et al.* 2017).

Although the importance of information-sharing was emphasised in our focus group discussions, some participants recognised the challenges described above:

*“So, you’ve got your people who know nothing and that think everything is terrible. You’ve got your people who know nothing who think everything is good. And all you do by telling them about what you’re doing is tell people that actually well, no, there are problems. So, the people who thought there weren’t problems, you just told them there are problems. And people who thought there were problems just reinforced because you wouldn’t be doing the research if there wasn’t a problem.”* [Veterinarian D2].

Others expanded on the idea that public attitudes are variable within populations, an idea consistent with previous research. For example, Meuwissen *et al.* (2007) distinguished six distinct animal welfare orientations among Dutch citizens, Vanhonacker *et al.* (2007) another six categories among Belgians, and Prickett *et al.* (2010) described three segments of American citizens. One participant in our study described the Prickett *et al.* (2010) categorisation of basic welfarists, naturalists, and price-seekers:

*There was some interesting research published a few years ago … they divided them into basic welfarists, the people … that wanted the animals to have better welfare but still be used for animal production. Purists, who specifically wanted animals to be in their natural environment, and then there was a small group that just wanted the product to be produced as cheaply as possible … And they really couldn’t push people out of these groups. If an individual didn’t want the animal to be used for agriculture, you aren’t going to change their mind … My point is … the conclusions may be drawn differently depending on our perspective.”* [Academic Veterinarian C7].

In line with recommendations to reimagine public engagement about science (Bucchi 2008; Simis *et al.* 2016), including animal welfare (Fernandes *et al.* 2019; Ventura & Fjæran 2021), some of our participants advocated for increasing partnerships with experts in the social sciences (e.g. *“I think we need to start looking at engaging people that study human behaviour”* [Academic Veterinarian C7]). One individual specifically called for the need to build relationships with external entities viewed as having public trust: “*So maybe we should work on providing our results in a meaningful way to the NGOs, hoping that they use their power they have on the consumer in a better way for us. And I’m totally aware that this is a very risky thing to do*” [Animal Scientist C3]. Others felt that education efforts were better focused within rather than outside the industry: “*I think you have to first educate the farmers and the veterinarians before the public … Why? Because we are the first actors in the field”* [Veterinarian A1]. Similarly, the Hendricks *et al.* (2022b) study of Canadian veterinarians discussing surplus calves found that most participants viewed their role as educating farmers (rather than the public).

### Theme 4: The public as helper or hindrance

All groups envisioned the public as involved in resolving cattle welfare issues to some extent. Conversation sometimes distinguished between the public’s role as *citizen* versus *consumer* (“…*citizens can agitate for direct action to change how animals are used through political processes, whereas consumers influence standards through their activities in the marketplace*,” (Degeling & Johnson 2015; p 963). Some of our participants expressly linked these two roles, e.g. “*Some of the public, they can be either consumers or they can be lobbyists. So, they are the people that buy the product, but they’re also the people that write letters to the government saying, ‘we want to ban this, we want to ban that’ … There are two roles, as I see it*” [Animal Scientist D1]. However, participants often expressed some ambivalence about one or both roles.

#### Public as citizens

Each group made specific mention of people exerting political pressure (e.g. voting on legislation and pressuring public officials). Much of this commentary was contributed by our Dutch participants, who described the public as already having a political effect due to the country’s welfare-focused NGOs and political party (Partij voor de Dieren [Otten & Gremmen 2016]). Some viewed this development as worrisome: *“Public opinion, politically at this time, it’s becoming an issue*” [Veterinarian B5]. For others, public opinion was viewed as motivating change: “*What we have in Holland is a strong NGO on animal welfare … and we also have an Animal Party in the parliament. So, they do raise a lot of those issues and because of the public opinion, they actually do get things changed”* [Animal Scientist D5].

#### Public as consumers

Many discussions also addressed potential or existing ways for the public to influence cattle welfare through their role as consumers. For example, Animal Scientist C9 explained how Swedish consumer pressure influenced decisions to avoid sale of Danish pork in the country due to discrepancies in tail-docking practices: “*Now what’s happening is that wholesalers and the individual grocery stores are putting a ban on Danish pork. Giving the consumers a good possibility to make a standpoint here in what they perceive as a welfare issue*,” and, “*I would agree with* [Academic Veterinarian C7]; [Retailers] *are already involved because they have their customers pushing them.”*

The focus groups also discussed if consumers were willing to pay (WTP) for products produced according to higher welfare standards, sharing their beliefs that consumer WTP for welfare would drive change on farms. While there is some evidence that Europeans are more willing to pay for animal welfare attributes compared to the world average (Cicia & Colantuoni 2010), market share for high animal welfare products remains limited (Harvey & Hubbard 2013). Nevertheless, many of our participants believed the consumer must pay more if improvements in welfare are to be achieved. This idea was voiced in several ways, e.g. *“… we need to arrive to the consumer because finally the consumer probably has to take some of these additional costs”* [Animal Scientist C2], *“In the end, they have to pay”* [Veterinarian E5]. Arguments also focused on WTP as a way for consumers to share the burden with farmers and for some as a way to honour the process of farming. In the words of Veterinarian E2: “*… I think the consumer* [has] *also to change his mind about food. He* [has] *to … have more respect for foods … It’s not only the farmer who has to work on animal welfare. It’s those consumers to have respect … and they have to pay for it.”*

Some participants suggested that consumers would indeed pay more; for example, Veterinarian A8 commented, *“I think they will pay maybe even more for an animal in good health, and a happy animal…”* while in another group Veterinarian D2 shared the perspective, “*you can go into the supermarket and you can buy four different brands of milk … you can see how much each one sells, and most people will buy the expensive milk.”* However, other participants expressed some uncertainty about WTP, e.g. “*Is the consumer ready to pay more? If he has the information of the origins of the animal welfare, the welfare status?”* [Veterinarian A5] and “*Belgium is in big discussions about ‘will the consumer* [pay] *… is he prepared to pay more for high standard milk?”* [Veterinarian E2]. Others were more straightforward in their pessimism: “*Most of the time the people decide for the cheaper meat and for the cheaper milk”* [Academic Veterinarian D4]. Veterinarian A7 took issue with WTP as a luxury unavailable to many, feeling that it was unrealistic to rely on that avenue to drive change:

*I disagree with you … we’re very lucky, veterinarians have… probably most of us have a reasonable lifestyle. But there are too many people who don’t have that lifestyle, who actually … you know, that five pence or a euro less than they pay for X, Y and Z is a huge amount for them. And I don’t think they even consider what the animal is doing or been through to produce that milk or how it’s been slaughtered or anything like that. I just don’t think it even registers on their radar. So, any change has got to be driven, I agree, the consumers have got to want it, got to be able to pay for it. But I think it’s naive to think that that is going to happen in the short term … I think there’s too many people in that bottom tier who just can’t.*

Participants also worried about the citizen-consumer gap, i.e. *“individual citizens who express an interest in farm animal welfare* [but who] *make consumer purchase decisions which do not reflect this*” (Vigors 2018; p 2):

*I feel the consumer has a very split personality. On one side he wants high quality products which have been produced under optimal animal welfare standards … on the other wise, he wants low price products all the time.* [Animal Scientist C3].

*They all want more welfare. But as long as there’s cheap meat offered they buy the cheap.* [Academic Veterinarian D4].

*That list changes depending on where they are. If they’re in a supermarket, you’d actually find it probably reshuffles slightly and price probably comes to the top … And then if they’re out reading a newspaper, in actual fact welfare comes to the top probably. Yeah, it’s just human nature, isn’t it?* [Veterinarian E3].

Some viewed this issue with pragmatism, stating that a gap between attitude and purchasing behaviour was normal (*“It’s similar like nobody is in favour of child labour in Europe. However, a lot of us have smartphones and we definitely know that those smartphones have been produced under extremely poor labour conditions in China”* [Animal Scientist C3]). It was unsurprising that this topic occupied so much discussion in some groups; as Aerts (2013) explained, *“It is clear that this situation proves a serious hurdle for* [especially] *European producers that are confronted with increased production prices, constant* [if not decreasing] *product prices, and import from countries with different* [if not lower] *production standards*” (p 172). Yet Aerts (2013) and others (de Bakker & Dagevos 2012) have questioned the citizen/consumer duality and its framing of the problem, suggesting that it rests on a false assumption. As Aerts (2013) states, “*Focusing on the consumer to drive the change is like talking to the person in the passenger seat*” (p 175). Instead, Aerts argued for a redirection of focus away from individual members of the public and toward retailers, a stance also advocated by some of our participants.

### Ambivalence about the public

Overall, we noted internal conflict among participants in their discussions about the public. Some expressed concerns about the power held by the public, sharing fear or uncertainty about the role the public could play in influencing farming from a place of ignorance, while also holding out hope that this power could bring about positive change if correctly harnessed (i.e. anticipated and shaped) by those in the veterinary and scientific communities. In the words of one participant, *“I think it’s better to anticipate that and to look at the systems we have and show how we’re aware of this welfare friendliness and trying to improve it, before it’s superimposed by the consumer. So, the consumers are very useful, but they’re also a little bit of a threat”* [Veterinarian B6]. Another participant referred to consumers as having *“a huge role, huge role”* but also that:

*The thing is though, we got to be ahead of that. We can’t let our – what we do to improve animal welfare be completely dictated by what the consumer is telling us, because … they don’t really understand completely. They’re not educated. We’re going down a road we don’t need to be going down. But they have a humungous role, a huge role, but we have to be ahead. We should take it on our own to make the changes that can satisfy them. That we think would satisfy. We have to listen to what they want, but then we have to… define. Sift through what they said and determine what’s the real key things we could – we should address.* [Veterinarian B2].

Ultimately, an overriding sense from these discussions was ambivalence rooted in a mix of frustration, fear, and hope, reflecting the diversity of perspectives about who the public is, whether what they want and know is legitimate, their roles in these issues, and how the cattle industries should respond to, or engage with, such concerns.

### Animal welfare implications

This study focused on the views of veterinarians and animal scientists in recognition of their roles influencing animal welfare on farms and in ‘bridge-building’ between diverse stakeholders within and external to the industries. Veterinarians, scientists and others holding advisory roles “*may be of more long-term use to the industry if they are able to navigate divergent views and help farmers achieve more socially sustainable practices*” (Ritter *et al.* 2021; p 7991). In the time since these participants shared their views, some work has shown that veterinarians do not perceive their role as defending traditional animal care practices (Hendricks *et al.* 2022b) but other work illustrates wide variation in veterinary attitudes towards cattle welfare (Canozzi *et al.* 2020). Continued research in this area is needed given the influence these stakeholders can have on animal welfare in the farming sectors.

We recommend increased attention to the training of animal sector stakeholders to include skills in communication and public engagement, to better allow a diversity of voices in conversations and decisions about agriculture. As stated by Ritter *et al.* (2021), “*an honest attempt to understand why members of the public consider specific welfare aspects important is substantially different from just trying to please lay members of the public who are perceived to lack agricultural knowledge; the latter mindset still portrays a lack of fully trying to acknowledge the validity of their views*” (pp 7991–7992). Democratising livestock production through the inclusion of public voices may nudge production practices into alignment with societal values (Guehlstorf 2008), improving public trust (see Rollin 2004; Bolton & von Keyserlingk 2021) and hopefully contributing to welfare improvements. We urge the livestock industries to seek out meaningful opportunities for partnership in which industry and society can together develop strategies for a more sustainable future.

## Study considerations and conclusion

This study sought to examine perspectives of veterinarians and animal scientists toward the general public’s concerns about and roles regarding cattle welfare. Our participants identified a range of issues including pasture access, restrictive housing, and painful procedures that prompt concern amongst members of the public. Many of our participants appeared to adopt the deficit model of public understanding, in that they believed that many of the public’s concerns were rooted in ignorance, and that with better educational efforts these concerns would resolve. However, it is important to note that participants were not unified in this belief and some also raised concerns about adopting deficit thinking. We also identified some ambivalence in participants’ attitudes to whether and how the public could contribute to improvements in cattle welfare. While much attention was paid to the public’s role as consumers, no clear consensus emerged about the efficacy, or indeed the desirability, of expecting consumers to shoulder this responsibility alone.

### Study limitations

Our use of focus groups provided veterinarian and animal scientist participants the opportunity to engage in deep discussions with colleagues in the cattle industries. This approach had the advantage of providing detailed, richly descriptive data on complex phenomena (Krueger & Casey 2009) and allowed us to capture the complexity with which participants perceived ‘the public’ role in discussions on cattle welfare. Our aim was not to generalise findings to the wider veterinary or scientific communities from which our participants were drawn as more quantitative approaches may seek to do (Carminati 2018); indeed, we caution against such generalisations and urge consideration of the results in context of our participants: for example, most participants were based in Europe and were male, and our recruitment at a meeting about farm animal welfare may have led to a focus on more progressive or informed stances. For these reasons we urge further research to consider additional cohorts of participants. We also note that the focus groups described here were conducted in 2014 and acknowledge that some perspectives of the participants may have changed; the Brexit referendum, the COVID-19 pandemic, the war in Ukraine, and cost of living crisis have shifted perspectives for both the livestock sectors and the general public in Europe and elsewhere. We encourage continued exploration in this area but highlight that the themes identified here resonate with findings from other work on industry stakeholder perspectives toward livestock welfare and the public (Buddle *et al.* 2021). Further, ours is one of relatively few qualitative studies that specifically prompted in-depth discussion of views of ‘the public’, revealing a rich and varied depiction from these stakeholders. Our questions asked focused on the views of veterinarians and animal scientists; we recommend future work address the views of other critical stakeholders, including retailers (e.g. Aerts 2013).

## Data Availability

Data are not publicly available to protect participant confidentiality with transcripts in the public domain.
